# Nurse-Patient Communication During Postpartum Discharge Teaching: Protocol for a Mixed Methods Study

**DOI:** 10.2196/72139

**Published:** 2025-10-17

**Authors:** Rebecca R S Clark, Patrina Sexton Topper, Tamar Klaiman, Rain Jacobson, Nadia Ngom, Naomi Kasahun, Kimberly De La Cruz, Celsea Tibbitt, Rebecca F Hamm, Milisa Manojlovich

**Affiliations:** 1 Center for Health Outcomes and Policy Research School of Nursing University of Pennsylvania Philadelphia, PA United States; 2 Pennsylvania Hospital Philadelphia United States; 3 M Louise Fitzpatrick College of Nursing Villanova University Radnor United States; 4 Perelman School of Medicine University of Pennsylvania Philadelphia, PA United States; 5 School of Nursing University of Michigan–Ann Arbor Ann Arbor United States

**Keywords:** nurses, postpartum period, communication, discharge planning, ethnography

## Abstract

**Background:**

Communication failures in inpatient maternity care are one of the leading causes of preventable maternal mortality. Most maternal mortality occurs during the postpartum period after hospital discharge. Nurses provide most direct inpatient maternity care and are responsible for postpartum discharge teaching, which is a critical moment for communicating about the care plan, concerns, warning signs, and follow-up plans to the patient, who will likely not be seen by a health care practitioner for 6 weeks, if at all.

**Objective:**

The purpose of this study is to develop a deeper understanding of communication practices between nurses and first-time mothers during postpartum discharge teaching, including what supports or hinders the transfer of critical information and recommendations for improvement from the nurses and patients themselves. A secondary objective is to assess the acceptability, feasibility, and appropriateness of video-reflexive ethnography (VRE) as an intervention to improve care quality and processes.

**Methods:**

We are using a health equity–informed mixed methods study design to develop a deeper understanding of communication practices between nurses and patients during postpartum discharge teaching for first-time mothers, including determinants for optimal communication and recommendations for improvement. Qualitative data will come from VRE, which will take place in 3 rounds: round 1 comprises video recording of actual postpartum discharge teaching, round 2 comprises independent review of the recording by both nurses and mothers, and round 3 comprises group reflexivity sessions with nurse participants. The planned analyses include a qualitative descriptive analysis of the video recordings and qualitative content analyses of the transcripts of the independent review and group reflexivity sessions. Quantitative data will come from a survey of nurse respondents regarding the feasibility, acceptability, and appropriateness of using VRE to reflect on and improve their practice. Survey results and reflections on VRE from round 3 will be integrated into a joint display.

**Results:**

This project was funded in 2023 and approved by the Institutional Review Board of the University of Pennsylvania on December 6, 2023. Data collection will take place from 2024 to 2025. Results are expected to be published in 2026.

**Conclusions:**

Our work aims to engage with nurses and first-time mothers to identify opportunities to improve postpartum discharge teaching and communication. Secondarily, we plan to find out whether study participants find VRE feasible, acceptable, and appropriate for improving the quality of care and health care communication.

**International Registered Report Identifier (IRRID):**

DERR1-10.2196/72139

## Introduction

### Background

The United States has the worst rates of preventable maternal morbidity and mortality of any high-income nation despite national efforts to reverse increasing trends in these rates [1]. Most maternal mortality (53%) occurs in the 7th to 365th day post partum, in the period during which a woman is typically home from the hospital [2]. Communication failures in inpatient maternity care are one of the leading causes of preventable maternal mortality in the hospital [3-14]. Postpartum discharge teaching is a critical communication moment with mothers immediately before they leave the hospital after giving birth and is often the last contact that a woman will have with a health care practitioner for 6 weeks, if not more.

Nurses provide most direct inpatient maternity care. Critically, nurses provide most, if not all, postpartum discharge teaching. This education typically includes information about warning signs, what to do and who to contact if problems arise, self-care, and the need for follow-up. Communication at this moment has massive potential—to help women and families know when to return for care to prevent morbidity and mortality and know what is normal to avoid unnecessary trips to seek care. Nurses and patients are the experts when it comes to the provision of and experience with postpartum discharge teaching.

Video-reflexive ethnography (VRE) is a method developed from health service research and quality improvement in health care settings to help frontline practitioners improve care processes and quality, including communication [15-18]. Theoretically, VRE is informed by complexity theory and exnovation, wherein the strengths of existing clinical practices are revealed to further improve care [19]. This method is unique in its capacity to be both an intervention to improve communication and a facilitator to advance equity. Regarding the former, watching oneself communicate is an evidence-based method for improving communication skills. Regarding the latter, watching a video of the implementation of an evidence-based intervention allows the viewers to build an understanding of what it is about the intervention or its implementation that supports or detracts from equity efforts [20]. VRE has similarities to the postgame video watching that sports teams engage in to determine strengths and learnings before their next game. For example, VRE has been used to improve nurse-physician communication in hospital settings [15], as well as communication with patients and families [18]. The limitations of VRE include wariness of being recorded on the part of clinicians and the amount of effort required to conduct it well. Wariness of being recorded seems to be most easily overcome by having clinicians champion the experience of VRE to their colleagues [21]. While the amount of effort required for VRE to be conducted well is unlikely to change, experience and expert mentoring can facilitate the process. VRE has been minimally used in the inpatient maternity setting [21,22]. Studies in this area have indicated that using VRE in inpatient maternity settings is generally feasible and acceptable [21]. This method is a means of facilitating care improvement, revealing where communication falls short of shared understanding, and helping participants become aware of patterns or aspects of their communication that they want to change [15].

### Objectives

The purpose of this study is to develop a deeper understanding of communication practices between nurses and first-time mothers during postpartum discharge teaching, including what supports or hinders the transfer of critical information and recommendations for improvement from the nurses and patients themselves. A secondary objective of this study is to understand the feasibility, acceptability, and appropriateness of VRE regarding improving nursing professional practice. The aims of this study are to (1) describe nurse-patient communication practices during postpartum discharge teaching; (2) determine barriers to and facilitators of effective communication during postpartum discharge teaching according to nurses and patients; and (3) determine the feasibility, acceptability, and appropriateness of VRE to improve nurse-patient communication during postpartum discharge teaching.

## Methods

### Overview

The overall strategy and methodology of this study is to use a health equity–informed [20] exploratory mixed methods design to develop a deeper understanding of communication practices between nurses and patients during postpartum discharge teaching for first-time mothers, including barriers to and facilitators (determinants) of optimal communication and recommendations for improvement from the nurses and patients themselves.

### Study Setting

This study will be conducted on the postpartum unit at Pennsylvania Hospital, an urban academic hospital that is part of a larger academic health system. The hospital, in addition to being the nation’s first, has the highest birth volume (approximately 5000 births per year) in Philadelphia, the city where it is located. The postpartum unit consists of roughly the same area on 3 hospital floors, one right over the other and connected via locked stairs (elevators allow visitors to access the different floors of the postpartum unit). The charge nurse typically assigns patients to each nurse on 1 of the 3 floors (there is 1 charge nurse per shift for all 3 floors). As part of an academic health system, the nursing staff engage in a wide array of quality improvement, evidence-based practice, and research projects. The city is highly diverse; approximately 30% of births annually are to Black women, and roughly 17% of births are to Asian and Latina women, with most of the rest being to White women.

### Design

This is a health equity–informed exploratory sequential mixed methods study designed to develop a deeper understanding of communication practices between nurses and first-time mothers during postpartum discharge teaching and, secondarily, to evaluate the use of VRE for optimization of postpartum discharge planning. This work is informed by the health equity implementation framework by Woodward et al [20], which centers the clinical encounter (or patient-health care provider interaction) as 1 of 3 critical moments affecting health disparities. This framework [20] also includes facilitation, which is how the evidence-based intervention is implemented for equity. The authors note that they do not focus on facilitation as the science in this area as the adaptation of the implementation process to promote equity is nascent. We believe that VRE has the potential to be not only an evidence-based intervention in and of itself but also a means of facilitation to promote improved care processes, outcomes, and equity.

**Figure 1:**
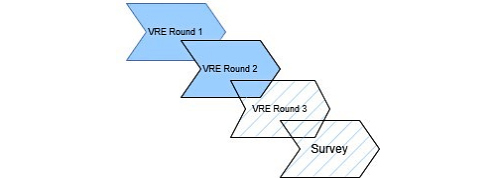
Overview of the study phases. Solid boxes include nurse and patient participants. Hatched boxes include nurses only. Overlap of boxes indicates that there can be overlap in the phases of the study (eg, not all round 1 recordings will be complete before round 2 interviews begin).

This exploratory mixed methods study involves VRE (qualitative data) followed by a survey (quantitative data) on VRE acceptability, feasibility, and appropriateness (Figure 1) [23]. VRE involves three rounds: (1) postpartum discharge teaching recording, (2) independent review of the round 1 recording by nurses and patients, and (3) reflexive review of round 2 findings by nurses in group sessions. Upon completion of the round 3 group reflexivity sessions, nurses will complete the survey. Both patients and nurses will complete a short sociodemographic questionnaire after their final session to collect information about race and ethnicity and years of nursing experience (for the nurses). For VRE, the planned analyses include a qualitative content analysis of the transcripts from the independent review and group reflexivity sessions with nurses. Coding for the qualitative content analysis will be both inductive and deductive (see the Analysis section for further details). For the survey, we will use descriptive statistics to report nurse responses regarding the feasibility, acceptability, and appropriateness of using VRE to reflect on their practice [23]. We will integrate the quantitative survey findings with the qualitative findings on the use of VRE from round 3 in a joint display. Table 1 provides further details.

**Table 1:** Data collection and analysis processes.

VRE^a^ round		Analysis process
**Before data collection**		
	Test whether the Owl cameras will sufficiently capture video and audio in postpartum hospital rooms.	—^b^
	Pilot video review checklist: face validity with postpartum nurse educator and observation of postpartum discharge teaching sessions to test the checklist and iterate as needed.	The postpartum nurse educator’s feedback was incorporated into the checklist. Two team members observed a postpartum discharge teaching session and spoke with the nurse about her process before incorporating her feedback.
	Conduct a technical “dry run” before proposed recording dates to understand logistical issues and plan accordingly.	—
**Round 1: video recording**		
	Nurses and patients will be consented before recording; we plan to oversample women from racial and ethnic minority groups.	—
	Video record postpartum discharge teaching between nurses and patients.	—
	The team views the video with the video review checklist (Multimedia Appendix 1).	Video-recorded postpartum discharge teaching will be evaluated and described according to the checklist (ie, presence or absence of clinical content, evidence-based teaching interventions, and effective communication techniques). Video clips will be chosen based on the presence or absence of something on the checklist through team consensus. The length and number of clips created may vary.
**Round 2: independent review**		
	Participants will be sent a link to a separate subfolder within Box with their selected video clips for confidentiality and may independently review these clips of their postpartum discharge teaching interaction during a recorded video call.	—
	Independent review will be conducted with a member of the study team (Multimedia Appendix 2). Participants will be encouraged to stop the video at any point and comment on their thoughts and feelings, including what they were thinking at the time (see the interview guide).	We will conduct qualitative content analyses of the transcripts of round 2 review sessions using both an inductive and deductive approach.
	Specific recommendations to improve the interaction and communication in general will be solicited at the end of the session.	—
	Patient comments will be edited into the video at the exact time stamp when they were made.	—
**Round 3: reflexivity sessions**		
	Nurses will be invited to participate in group reflexivity sessions that will be held virtually.	—
	We will ask participants to reflect on round 2 clips that have embedded patient comments (see the interview guide).	Transcripts of the reflexivity sessions will be analyzed using the qualitative content analysis described in round 2.

^a^VRE: video-reflexive ethnography.

^b^The left-hand column includes a step in the data collection process for which analysis was not applicable.

### Eligibility Criteria

Nurses are eligible for recruitment if they are postpartum nurses at the study hospital and conduct discharge teaching. Patients are eligible for recruitment if they are first-time mothers being discharged with their infant, are aged older than 18 years, and speak English. We chose to limit eligibility to those aged older than 18 years because emancipation is not automatically granted to minors who become pregnant in the state where the study is being conducted. We also chose to limit eligibility to patients who spoke English due to the additional complexity that translation adds to a clinical interaction.

### Ethical Considerations

This study received approval from the University of Pennsylvania Institutional Review Board (IRB) before initiation of data collection (December 6, 2023; protocol 854936). Informed consent was obtained from all participants (nurses and patients). Data in all research products, including findings shared with the nursing staff and leadership, will be deidentified. Participants received monetary compensation via a Greenphire ClinCard for taking part (US $60 for completion of round 1 and US $50 each for completion of rounds 2 and 3).

### Researcher Characteristics and Reflexivity

The principal investigator of this study is the nurse scientist at Pennsylvania Hospital, and before her work as an assistant professor, she practiced clinically as a nurse and then as a midwife. Her clinical practice informs her research questions and assumptions, as does her work as a maternal health service researcher focused on excellence and equity in quality and outcomes. Her background is part of what made VRE as a method so attractive because she recognizes the importance of patient and frontline providers’ perspectives and expertise for improving care and outcomes. The research team, which has a history of successful collaboration, involves people with a wide array of lived experiences and sociodemographic diversity. Having so many people on the team coming from different points of view helps balance assumptions and presuppositions. Finally, one of our team members is a nurse from the postpartum unit where the work is being conducted. As a nurse champion, her presence and expertise on the team has had a positive impact on recruitment and will inform data analysis.

### Sample Size and Recruitment Plan

The sample will consist of postpartum nurses who work in a large urban hospital and the new mothers who receive education from them before discharge. There are 86 nurses in the unit, and approximately 5000 women give birth in the hospital annually. We are looking to record 20 dyads (1 nurse and 1 patient; 40 total participants) for VRE round 1. Previous VRE research conducted elsewhere has reported recruitment rates of 75% of nurses [15]. To recruit nurses, we will (1) present the study at unit council meetings to get the word out and establish buy-in before speaking with individual nurses, (2) post recruitment flyers in nurse locker rooms and send reminder emails about the study, and (3) seek the collaboration of nurses who are informal leaders and unit champions to support recruitment efforts. At the unit council meetings, we will share information about the work, gather input, address concerns, and answer questions. We are also seeking to understand the following: the best times of day for video recording, optimal placement of recording equipment in different room configurations, and how nurses will know when a mother is about to be discharged (how much lead time the nurse will have). We hired one of the postpartum nurses to be a research team member. This nurse will be either a formal or informal leader in the unit and, in addition to sharing their clinical and contextual expertise with the research team, will receive training to consent participants and record postpartum discharge teaching. They will also participate in data analysis, interpretation, and dissemination. After attending unit council meetings, we will spend time in the unit talking with individual nurses and inviting them to participate.

Patient participants will be recruited on the day of discharge based on the assignments of nurse participants who are working on any given day. We will also place study flyers in shared lounge spaces on the postpartum unit.

### Data Collection and Management

To collect data, we will use Owl cameras (a smart webcam designed with a 360° camera and microphone that becomes smarter over time; Owl Labs) set up on tray tables that will be wheeled into patient rooms before the beginning of the postpartum discharge teaching session. The Owl cameras interface with a videoconference platform (we will use Zoom [Zoom Video Communications]) to create a video and transcript. Audio and video of the meeting will be recorded to a secure research drive (the HIPAA [Health Insurance Portability and Accountability Act]-compliant Box). We are specifically recording to Box versus saving to Zoom and then transferring data to enhance data security. Table 1 presents both the data collection and management and analytic processes. Before collecting data, we will pilot a video review checklist at postpartum discharge teaching sessions. The video review checklist was initially developed based on existing recommendations for postpartum discharge teaching [22,24-26] and clinical expertise from the team. We then established face validity for the checklist by having the postpartum nurse educator review the checklist and pilot-test it during a postpartum discharge teaching session that was not part of the research. We will also have “dry run” days on the unit in which the research team will observe the postpartum nurses’ workflow over the course of the day to understand how best to run the logistics of the study and test the equipment. We will also examine the postpartum discharge rooms to determine the best placement for the Owl cameras.

Postpartum discharge instructions are typically delivered in the late morning or early afternoon on the day of discharge. We will check in with the charge nurse shortly after shift change to ask whether any of the nurse participants working that day have a first-time mother being discharged. If they do, the study team member will approach the patient to invite them to participate in the study. If the patient agrees, the study team member will speak with the nurse about when they plan to conduct discharge teaching and proceed with setting up the recording session using the Owl camera.

The study team member will place an Owl camera in the room in a predetermined optimal position, make sure that it is on and recording to the Box folder, and that the setup is acceptable for the nurse and patient before leaving the room. After postpartum discharge teaching is complete, the study team member will collect the Owl camera and check to ensure that the team has the preferred contact method for each participant to schedule the round 2 interview.

VRE round 2 interviews will be conducted virtually via Zoom. In these sessions, nurses and patients will independently review the postpartum discharge teaching recording that they participated in and reflect on what went well and what might be done differently. Participants will be encouraged to reflect on what they were thinking during particular moments of the interaction, as well as on the experience as a whole. The interviews will be transcribed using the autotranscribe feature on Zoom and will again be saved to a secure research drive. Transcripts will be checked for quality by a trained research assistant. At the end of the round 2 interviews, patient participants will be asked to complete a short sociodemographic survey in REDCap (Research Electronic Data Capture; Vanderbilt University). Round 2 interviews will be processed for round 3. Patient comments from round 2 interviews will be inserted into the appropriate point in the round 1 video (eg, the patient comments on a particular interaction or thing said in the round 1 video). Patient comments from round 2 that are not tied to a specific moment in the round 1 video will be kept separate. The team will select topics to present to the nurses during round 3. To do this, the team will watch the round 1 and round 2 videos to identify themes that arise from the data, as well as for places where data adhere to or deviate from the codebook. The selection of themes will be iterative and evolving as round 3 will begin before round 2 ends and feedback from early round 3 sessions may inform future round 3 sessions (eg, probing deeper on a particular topic).

VRE round 3 (reflexivity sessions) will be conducted virtually with nurses only by the same team members who conducted the round 2 interviews. Nurse participants (2-5 per session) will watch clips from round 2 that are organized by theme. The themes will be selected deductively (eg, based on the framework by Woodward et al [20] or the video review checklist from round 1) and inductively by the research team, with consensus on themes to include being reached through discussion. The process of reviewing the videos and determining themes will be ongoing during round 2 but finalized before round 3 begins. The nurses will be encouraged to reflect on their postpartum discharge teaching practices as a group and offer recommendations to improve this teaching and nurse-patient communication to their colleagues. These sessions will be recorded as mentioned previously. Recordings and transcripts will be managed as described previously as well. At the end of round 3, nurses will be administered 2 surveys (via REDCap) to share sociodemographic data and rate the feasibility, acceptability, and appropriateness of VRE as an intervention to improve nurse-patient communication using 3 validated short scales (Multimedia Appendix 3) [27]. We provided the funding organization’s review comments (Multimedia Appendix 4) and followed the Standards for Reporting Qualitative Research Guidelines in developing the manuscript (Multimedia Appendix 5).

### Analysis

Table 1 provides details on study procedures and analysis. As described in Table 1, round 1 videos will be analyzed by comparing them to the video review checklist (Multimedia Appendix 1). For rounds 2 and 3 (Multimedia Appendix 2), we will conduct qualitative content analysis using a deductive and inductive approach [28-30]. The deductive codebook will be based on the health equity framework by Woodward et al [20] and the video review checklist. Initially, the entire team will review the round 2 interviews of 2 dyads (4 interviews total) to develop initial inductive codes and discuss the application of the deductive codes. A process of abstraction [30] will be used to condense similar codes into descriptive categories and subsequent themes. Themes will be developed iteratively, and the team will collectively define final themes and subthemes until thematic sufficiency is reached [31]. We will aim to reach for 100% agreement across the analytic sample. After that, the interviews will be divided between 2 groups comprising 2 team members each, who will proceed with coding. Questions or disagreements will be brought back to the full group during weekly meetings. The team members will practice data immersion by watching available videos from rounds 1, 2, and 3 and by reading the interview texts to gain familiarity with the scope and depth of responses. This process will be completed for the round 2 interviews first, followed by the round 3 sessions. Descriptive statistics will be used to report the survey data and sociodemographic information. We will present findings from the acceptability, feasibility, and appropriateness surveys in a joint display with qualitative findings related to the nurses’ perspectives on the use of VRE. We plan to use ATLAS.ti (Scientific Software Development GmbH) and Stata (StataCorp) [32] to conduct the analyses. This study received IRB approval.

The research team will keep an audit document to track their analytic decisions and will debrief to discuss interpretive biases. We will incorporate the following techniques to increase the trustworthiness and rigor of our qualitative research: (1) development of standard operating procedures for recruitment, recording, and analysis; (2) prolonged engagement with the data; (3) audit trail maintenance to record the team’s analytic decisions; (4) extensive memos and field notes to track theoretical and reflective thoughts; (5) engagement in researcher triangulation and peer debriefing; and (6) full of context [33].

## Results

This study was funded in July 2023, although final approval for fund disbursement was received in February 2024. We received IRB approval in December 2023. Data collection will take place from July 2024 to December 2025. Results are expected to be published in 2026. To date, we have recruited 16 nurses and 10 patients. A total of 10 round 1 recordings been completed, and the follow-up round 2 independent reviews are in process as of this writing.

## Discussion

### Expected Findings

We expect that the main finding of this study will be nurse- and patient-generated opportunities to improve postpartum discharge teaching for first-time mothers. Furthermore, we expect to find some content and communication style variation in the postpartum discharge teaching that nurses provide to first-time mothers (round 1). We further expect that both nurses and patients will have observations as to what went well and what might be improved (round 2) and that the nurses—upon reflection as a group (round 3)—will have recommendations to improve these critical communication moments. This work is the foundational step in not only understanding how to improve postpartum discharge teaching but also building capacity to use VRE as a critical means of improving communication in maternity care (as communication failure is a leading root cause of preventable maternal morbidity, mortality, and racial disparities therein). Prior VRE work in general has been predominantly conducted outside of the United States. The VRE work conducted in the United States, while highly formative, has not been conducted in a maternity setting [15,17,18]. This is the first instance of VRE being conducted in a maternity setting in the United States of which we are aware. Other VRE work in maternity spaces has been conducted worldwide and proven highly informative (eg, suggesting that VRE was feasible and acceptable in a maternity setting in the United Kingdom [21,22]).

There are strengths and limitations to this work. Video recording clinical interactions is a sensitive subject. To address this, we have met with clinical nursing leadership at the hospital, who have confirmed with the research team that the videos will not be used to penalize nurses. We will ask for nurses’ recommendations about how to address perceptions of risk of being penalized for future participants (eg, what circumstances might make participation feel like less of a risk and their suggestions to address risk). As a result, findings will only be shared with the leadership team as a deidentified aggregate. We will hire at least one postpartum nurse to be a study team member who will become a familiar presence during the study. This idea was endorsed by the clinical director and unit leadership. The nurse on the research team will not be a study participant. Other interested nurses will be encouraged to champion the work to their peers.

The Hawthorne effect is frequently mentioned as a concern. However, previous research indicates that many participants eventually stop paying attention to the recording device in the room [34-36]. To address this challenge, we will use an Owl camera, which has a small and less obtrusive footprint. We will also ask participants about their awareness of being video recorded as part of the postvideo survey. Prior work has found that it is important to minimize the time gap between initial video recording and independent review [15]. To address this, we will schedule independent reviews (round 2) with patients and nurses when their interaction is recorded with the goal of having that round 2 meeting within 3 weeks for nurses and 4 months for patients. The longer time frame allows for flexibility in scheduling for first-time mothers.

To assess the feasibility of this work, we engaged clinical leadership both as team members and as advocates for the project. With regard to ongoing feasibility, we will ask nurse participants to complete a feasibility survey as part of the postrecording data collection.

Even with these limitations, the planned study has numerous strengths. First, we are submitting a protocol as a point of rigor for the work. Second, VRE is a powerful (and underused in the United States) methodology for studying and improving clinical practice. Third, we have a stellar team representing contextual, clinical, and research experience and expertise, including VRE.

Future directions for this work include the development of evidence-based interventions to improve aspects of postpartum discharge teaching identified in this work. Other directions include the use of VRE to improve other aspects of health care communication in the inpatient maternity setting, perhaps as identified in other work based on incident reports [37].

### Dissemination

Upon completion of this work, we plan to share the results back to the postpartum unit at the participating hospital during a unit council meeting and any further meetings recommended by the clinical and operational leadership. We will also offer to share the paper with the nurse and patient participants if they are interested. We will consider sharing this work at system-wide meetings as well. We will also disseminate our findings via conference abstracts and papers, with a plan to include the postpartum nurse hired to be part of the research team as a copresenter at one conference at minimum and as a coauthor on any papers. We plan to use the Systematic Development of Standards for Mixed Methods Reporting in Rehabilitation Health Sciences Research guide for reporting [38].

### Conclusions

Communication failure is a leading root cause of preventable maternal mortality. We are beginning to focus on the postpartum period, especially after hospital discharge, as a critical period that is underserved, underresourced, and understudied. Nurses provide essential education for patients and their families before discharge after giving birth. This study will serve to advance our understanding of communication practices between nurses and first-time mothers during postpartum discharge teaching, including what supports or hinders the transfer of critical information and what improvements to the discharge education process nurses and patients recommend. This work has the potential to inform an array of future work, including an implementation science study of (1) incorporating evidence-based information and communication techniques into postpartum discharge teaching and (2) VRE as a method to improve critical communication (and equity therein) in inpatient maternity care (eg, handoff from labor and delivery to the postpartum unit).

## Appendix

Multimedia Appendix 1 Video review checklist.

Multimedia Appendix 2 Round 2 independent video review guide.

Multimedia Appendix 3 Survey instrument.

Multimedia Appendix 4 Response to funder's comments.

Multimedia Appendix 5 SRQR checklist.

Multimedia Appendix 6 Peer review report by Betty Irene Moore Nurse Scholars and Leaders Fellowship Review Committee, UC Davis Health.

## Abbreviations

HIPAA: Health Insurance Portability and Accountability Act
IRB: Institutional Review Board
REDCap: Research Electronic Data Capture
SRQR: Standards for Reporting Qualitative Research
VRE: video-reflexive ethnography

## Data Availability

The datasets generated or analyzed during this study are available from the corresponding author on reasonable request.
