# Supplementary material for: Nurse-Patient Communication During Postpartum Discharge Teaching: Protocol for a Mixed Methods Study
Source: JMIR Res Protoc. 2025 Oct 17;14:e72139. doi: 10.2196/72139 (PMC12579284; doi:10.2196/72139)
Supplement: Multimedia Appendix 1 [file resprot_v14i1e72139_app1.docx]

| **Clinical Content** | Yes | No | NA |
| --- | --- | --- | --- |
| Reviews and confirms understanding of physical needs: |  |  |  |
| Summary of birth events (late preterm – special considerations; reviewed baby care) |  |  |  |
| Physical recovery and how to manage (driving, lifting less than 10#) |  |  |  |
| Incision care if appropriate – mention shower to keep incision clean? |  |  |  |
| Lactation / Breastfeeding |  |  |  |
| After pains (reviewed pain medication) |  |  |  |
| Activity recommendations – covered in physical recover |  |  |  |
| Nutrition (eat well, drink lots of water) |  |  |  |
| Urination/moving bowels |  |  |  |
| Medication use |  |  |  |
| Reinforcement of the value of outpatient postpartum visits |  |  |  |
| Who to contact with medical health concerns (see below) |  |  |  |
| Reviews and confirms understanding of gynecologic needs: |  |  |  |
| Perineal care |  |  |  |
| Lochia |  |  |  |
| Menstrual cycle/family planning |  |  |  |
| Sexual health and activity |  |  |  |
| Reviews and confirms understanding of mental health needs: |  |  |  |
| Emotional well-being |  |  |  |
| Sleep / fatigue |  |  |  |
| Self-care |  |  |  |
| Who to contact with mental health concerns |  |  |  |
| Review of maternal warning signs/symptoms (POST-BIRTH) including what conditions they might be related to, allowing for advocacy if an approached provider is not obstetrical or of another clinical specialty |  |  |  |
| Call 911 for ​​Pain in chest |  |  |  |
| Call 911 for Obstructed breathing or shortness of breath |  |  |  |
| Call 911 for Seizures |  |  |  |
| Call 911 for Thoughts of hurting yourself or someone else |  |  |  |
| Call your healthcare provider for Bleeding, soaking through one pad/hour, or blood clots, the size of an egg or bigger |  |  |  |
| Call your healthcare provider for Incision that is not healing |  |  |  |
| Call your healthcare provider for Red or swollen leg, that is painful or warm to touch |  |  |  |
| Call your healthcare provider for Temperature of 100.4°F or higher |  |  |  |
| Call your healthcare provider Headache that does not get better, even after taking medicine, or bad headache with vision changes |  |  |  |

| **Effective Communication** | Yes | No | NA |
| --- | --- | --- | --- |
| Appropriate verbal communication / conversational conduct: |  |  |  |
| Introduces themselves by name and role (if applicable) |  |  |  |
| Introduces new people in the room by name and role, explaining what they will do (if applicable) |  |  |  |
| Uses plain language. (Speaks without using medical jargon) |  |  |  |
| Demonstrates active listening skills. (e.g., I hear you say “XXXX”. Did I understand you correctly/Did I get that right?) |  |  |  |
| Focuses on the patient's and family's interests. |  |  |  |
| Thanks patient or family for calling attention to any issue raised. |  |  |  |
| Asks about and listens to the patient’s and family’s needs and concern |  |  |  |
| Invites them to continue asking questions. |  |  |  |
| Uses open-ended questions. |  |  |  |
| Understands when to seek additional support and help during a conversation with a patient and/or family |  |  |  |
| Appropriate nonverbal communication: |  |  |  |
| Makes eye contact with the patient and family |  |  |  |
| Smiles when appropriate |  |  |  |
| Shoulders are turned toward the patient |  |  |  |
| Shoulders “lean in” or deviate forward from the vertical plane (shoulders must be turned towards patient) |  |  |  |
| Torso (chest to waist) is open to the patient (shoulders must be turned towards patient) |  |  |  |

| Smile | Corners of the mouth are turned up and cheeks move up |
| --- | --- |
| Eye gaze | Nurse is looking directly at the patient |
| Orientation | Nurse's shoulders are turned toward the patient |
| Lean in | Nurse's shoulders deviate forward from the vertical plane (cannot lean if not oriented) |
| Openness | Nurse’s torso (chest to waist) is open to the patient (cannot be open if not oriented) |
