# Supplementary material for: Nurse-Patient Communication During Postpartum Discharge Teaching: Protocol for a Mixed Methods Study
Source: JMIR Res Protoc. 2025 Oct 17;14:e72139. doi: 10.2196/72139 (PMC12579284; doi:10.2196/72139)
Supplement: Multimedia Appendix 2 [file resprot_v14i1e72139_app2.docx]

Independent Review Guide

Thank you for joining us for the independent review! The way this works is that you are the expert, and

we are here to learn about the postpartum discharge teaching process. We will be watching clips of the

discharge teaching together. We would like to ask you to pause the video whenever you want and share

with us your observations about what is happening in a particular moment (what is going well, what you

are thinking or feeling, any observation you might have). We may also pause the video to ask you about

what is happening in that moment for our own understanding. What questions might you have before

we get started?

[At the end of the session, if participant is a patient, administer the survey. For either nurse or patient

participant, make sure ClinCard payment is set up. For nurse participant, make sure contact details are

correct and discuss focus group follow-up.]

What questions might you have for us at this time?

Thank you so much for your participation!
