# Supplementary material for: Nurse-Patient Communication During Postpartum Discharge Teaching: Protocol for a Mixed Methods Study
Source: JMIR Res Protoc. 2025 Oct 17;14:e72139. doi: 10.2196/72139 (PMC12579284; doi:10.2196/72139)
Supplement: Multimedia Appendix 3 [file resprot_v14i1e72139_app3.docx]

**APPENDIX B**. **SURVEY QUESTIONS**

1. Are you a patient or a nurse?

- patient

- nurse

2. Please provide your age

______ (years)

3. Please provide your race.

- Black/African American

- White

- American Indian or Alaska Native

- Asian

- Native Hawaiian or Other Pacific Islander

- Other race (Please specify)

4. Please provide your ethnicity.

- Hispanic

- Non-Hispanic

Video-reflexive ethnography (VRE) is the process of having an interaction videorecorded, watching it and reflecting on the interaction independently, and then as a group

**Acceptability of Intervention Measure (AIM)**

|  | Completely disagree | Disagree | Neither agree nor disagree | Agree | Completely agree |
| --- | --- | --- | --- | --- | --- |
| 1. VRE meets my approval. | ➀ | ➁ | ➂ | ➃ | ➄ |
| 2. VRE is appealing to me. | ➀ | ➁ | ➂ | ➃ | ➄ |
| 3. I like VRE. | ➀ | ➁ | ➂ | ➃ | ➄ |
| 4. I welcome VRE. | ➀ | ➁ | ➂ | ➃ | ➄ |

**Intervention Appropriateness Measure (IAM)**

|  | Completely disagree | Disagree | Neither agree nor disagree | Agree | Completely agree |
| --- | --- | --- | --- | --- | --- |
| 1. VRE seems fitting. | ➀ | ➁ | ➂ | ➃ | ➄ |
| 2. VRE seems suitable. | ➀ | ➁ | ➂ | ➃ | ➄ |
| 3. VRE seems applicable. | ➀ | ➁ | ➂ | ➃ | ➄ |
| 4. VRE seems like a good match. | ➀ | ➁ | ➂ | ➃ | ➄ |

**Feasibility of Intervention Measure (FIM)**

|  | Completely disagree | Disagree | Neither agree nor disagree | Agree | Completely agree |
| --- | --- | --- | --- | --- | --- |
| 1. VRE seems implementable. | ➀ | ➁ | ➂ | ➃ | ➄ |
| 2. VRE seems possible. | ➀ | ➁ | ➂ | ➃ | ➄ |
| 3. VRE seems doable. | ➀ | ➁ | ➂ | ➃ | ➄ |
| 4. VRE seems easy to use. | ➀ | ➁ | ➂ | ➃ | ➄ |

The AIM, IAM, and FIM are made publicly available in the following presentation: Weiner, B. J., Lewis, C. C., Stanick, C., Powell, B. J., Dorsey, C. N., Clary, A. S., Boynton, M. H., & Halko, H. (2017). Psychometric assessment of three newly developed implementation outcome measures. *Implementation Science*, *12*(1). https://doi.org/10.1186/s13012-017-0635-3
