# Supplementary material for: Nurse-Patient Communication During Postpartum Discharge Teaching: Protocol for a Mixed Methods Study
Source: JMIR Res Protoc. 2025 Oct 17;14:e72139. doi: 10.2196/72139 (PMC12579284; doi:10.2196/72139)
Supplement: Multimedia Appendix 4 [file resprot_v14i1e72139_app4.pdf]

Comment	Response
Refer to the tracked changes/comments included in the attached document	Tracked changes accepted. Please see responses to comments in the document.
Please align aims and methods	Please see second paragraph in the Design and Methods Section.
In regards to the Health Equity Implementation Framework guiding this work, the study focuses on the provider and patient recipients (and feasibility/acceptability at the dyad level), and none of the org-level/ contextual factors or other domains highlighted in the framework that would support overall implementation within a healthcare setting. As such, rather than stating the study design is geared for implementation, we think it is important to acknowledge your focus on providers/patients as two recipient groups and frame in terms of establishing an effective/feasible/acceptable approach to improve provider/patient communication – noting this is a first step – and the importance of future studies to expand the focus to include org-level considerations, and more expansive group of stakeholders (both directly and indirectly positioned to influence the success of implementation of VRE as an intervention within a healthcare setting)	Edited accordingly.
We recommend considering an expanded scope to include perspectives beyond the nurse and patient involved in the encounter, including perspectives about VRE as an educational approach to improve nurse/patient communication and org factors to consider in terms of successful VRE implementation as an intervention in their setting. For example, org-level leaders/admin, staff development/professional development leader and clinical leader perspectives about the potential utility of this approach. Perhaps you could present the findings to them and get input.	We do plan to present the findings to organizational leaders, administrators, and managers and get their feedback – and agree that this is important for next steps. I am concerned about formally expanding the study and my capacity to complete this in addition to what's already planned.
Phase 3 analysis: table indicates constant comparative analysis which differs from the	Thank you – edited for consistency.

narrative in sections that follow that indicates qualitative content analysis. Please clarify and be consistent throughout.	
Please clarify status of attaining organization- and dept-level approvals to conduct this study	Updated readiness to launch accordingly.
Consider implications of hiring two postpartum nurses (peers, informal/formal leaders on the unit) to be study team members in this sensitive process – perceptions of prospective nurse participants re: their colleagues/leaders viewing the videos and analyzing the recorded interactions? Have you explored the implications of this with practicing nurses? What is your thinking about this?	Please see final paragraph of Section 4. Was planning on talking with the nurses about this; research mentor's sense is to proceed with hiring one of the Mother-Baby Unit nurses and a research coordinator from the Women's Health Research Center.
Beyond voting, for 'moments'/video clips, be more explicit about how you're evaluating the videos. There are videos out there about quality of communication – this has been done a lot, empathy, listening, etc. - say more about how you're looking at those.	We will look for moments where teaching is personalized or seems generic, for the presence of medical jargon, and for particular moments when warning signs and symptoms are described (and whether these include mental health warning signs), and whether or not infant care is covered. We will look for the presence or absence of teach back and whether/how questions are solicited/encouraged, including moments when shared decision-making occurs or there are moments of assertion-acquiescence. We will also look for specific cues such as making eye contact, repeating back information (without being asked to), agreeing or disagreeing, body language that would suggest openness to what is being said, or alternatively discomfort, etc. Please see Table 1 for details added.
Clarify if nurses receive their individual video clips or aggregate?	Participants will watch their individual video clips independently in Phase 2 and will watch a selection of clips jointly with other participants in Phase 3. Further detail was added to the data collection table.
Please provide methodological rationale for approach (nurses/pts reviewing video clips...) - we thought you were going to take patient viewing	See first paragraph of Section 4, Design and Methods. We believe that patient reflections are valuable and decided that it should be their choice whether they want to continue in the study. .

out - what might be consequences for patient to revisit a non-supporting interaction?	
How do you see the intervention being used in the future? (staff development training, etc?)	We think the postpartum nurses may recommend changes to how education is delivered on their unit based on what they discover while participating in the research. This might include training on how to conduct postpartum discharge education or therapeutic communication for various cultural groups. It could also include tool development or a checklist to make sure nurses cover all necessary information. The nurse manager also thought that next steps might include sharing videos of patients' individual discharge teaching with them so that patients can review what the nurse said after they go home. I could also see this intervention being rolled out to other nurses on the unit, throughout the women's health service line, other unit types, or other groups of providers.
With regards to ongoing feasibility, and "the risk of being penalized – whether as a nurse by one's supervisors and peers, or as a patient by one's healthcare team.", consider including questions that seek recommendations for how to address risk perceptions moving forward – for those that decline participation or participate but are worried, consider asking them what circumstances might make it feel like less risk, their suggestions for how to address this risk	Great question. We will ask this of the participants. We can also consider surveying the non-participants about risk informally at a staff meeting. Added to Data Collection Activities paragraph of Section 4.
Previous comments not addressed:<br>- Phase 1 video recording: say more about the "member checking" component - what information will you be asking for as part of this activity?<br>- Phase 1 analysis: the communications of interest focus on communication gaps – consider the value of also ID'g communications excerpts that might reflect effective communication	My apologies for missing this!<br>- Regarding "member checking" in Phase 1, we will be asking them whether they are comfortable with the video moving forward for analysis, giving the participants the authority/power to say the video isn't going forward.<br>- Agreed! Added explicitly to Data collection table.
