# Supplementary material for: Nurse-Patient Communication During Postpartum Discharge Teaching: Protocol for a Mixed Methods Study
Source: JMIR Res Protoc. 2025 Oct 17;14:e72139. doi: 10.2196/72139 (PMC12579284; doi:10.2196/72139)
Supplement: Multimedia Appendix 6 [file resprot_v14i1e72139_app6.pdf]

UCDAVIS HEALTH | Betty Irene Moore Fellowship for Nurse Leaders and Innovators

Research Plan

(Use this template with the embedded format for margins and font size.
A maximum of 8 pages for the narrative (not including Tables and References).

Project Title Listen to Her: Communicating to Improve Racially Disparate Birth Outcomes
(no more than 200 characters long, including the spaces between words)

Fellow Name: _Rebecca Clark____ Date submitted:___________

1. Specific Aims (no more than 1 page)

The United States (U.S.) has the worst preventable maternal morbidity and mortality rates of any industrialized nation despite national efforts to reverse increasing trends in these rates.[1]~~[1]~~ Most (53%) of maternal mortality occurs in the 7th to 365th day postpartum, i.e., after hospital discharge.[2]~~[2]~~ Black women bear the brunt of preventable maternal mortality in the U.S., regardless of health or social risk factors.[2,3]~~[2,3]~~ Black women's experience of communication with maternity healthcare teams is reported as poor and marked by disrespect and a lack of listening.[4–6]~~[4–6]~~ Communication failures in inpatient maternity care are one of the leading causes of preventable maternal mortality.[4,5,7–16]~~[4,5,7–16]~~ Communication quality and effectiveness varies among healthcare teams, but there is evidence of more negative communication when the patient is Black.[17]~~[17]~~ Numerous interventions and strategies exist to improve communication in U.S. inpatient maternity settings.[18–20]~~[18–20]~~ These interventions, however, do not address racially disparate communication, nor have most of them been tested against patient outcomes. Designing, implementing and evaluating effective interventions to improve racially disparate communication is likely to result in decreased maternal morbidity and mortality in the U.S

Nurses provide most direct inpatient maternity care. Communication – the building of shared understanding – is critical to this care and encompasses such things as decision-making, education, and emotional support. Nurses are crucial communicators within the healthcare team and between the team and patient ~~and,~~and are ideally situated to disrupt – or perpetuate – racially disparate communication. Further, nurses typically provide most, if not all, postpartum discharge education, which includes information about warning signs, what to do and who to contact if there are problems, self-care, and the need for follow-up. Therefore, nurses are a critical population with whom to begin in considering how to effectively intervene to improve inpatient maternity communication.

Video-reflexive ethnography (VRE) is a method used in an array of disciplines to help people develop self-awareness and improve their communication patterns, including those which are racially disparate.[21–24]~~[21–24]~~ It has been minimally used in the inpatient maternity setting.[25]~~[25]~~ This method has the potential to be an intervention itself, revealing where

Formatted: Font: (Default) Arial, Font color: Black, Superscript

Formatted: Font: (Default) Arial, Font color: Black, Superscript

Formatted: Font: (Default) Arial, Font color: Black, Superscript

Formatted: Font: Font color: Black, Superscript

Formatted: Font: Font color: Black, Superscript

Formatted: Font: (Default) Arial, Font color: Black, Superscript

Formatted: Font: (Default) Arial, Font color: Black, Superscript

Commented [fl1]: I don't think you understood my comment on the prior version.

Commented [RC2R1]: Sorry that I didn't understand! Thank you for the continued revision!

communication is falling short of shared understanding, and helping participants become aware of patterns or aspects of their communication that they want to change.[21]~~[21]~~ As such, VRE may represent an innovative intervention to improve inpatient maternity communication overall, and disparate communication in particular. The purpose of this study is to develop a deeper understanding of communication practices between nurses and patients during postpartum discharge teaching for first-time moms, including barriers and facilitators to communication, and recommendations for improvement. Aims of this 3-year qualitative study using video reflexive ethnographic methods are: Aim 1: Describe nurse-patient communication practices, including variation by racial concordance, during postpartum discharge teaching. Aim 2: Determine barriers and facilitators to optimal communication during postpartum discharge teaching according to nurses and patients. Aim 3: Determine feasibility, acceptability, appropriateness of VRE to improve nurse-patient communication during discharge postpartum teaching.

The current study will ~~provide preliminary findings to~~inform an ~~support an~~ R01 responsive to PAR-22-064 (“Patient-Clinician Relationship: Improving Health Outcomes in Populations that Experience Health Care Disparities”). The call supports innovative, multi-disciplinary and multi-level research to build understanding as to how optimizing patient-clinician communication affects health care outcomes in patients from groups experiencing healthcare disparities. This includes identifying best practices and interventions that build and improve patient-clinician relationships leading to improved health outcomes and increased equity. ~~that either focuses on 1) the scaling and implementation of VRE to improve inpatient maternity communication or 2) the implementation of an evidence-based intervention identified in Aim 2.~~

Commented [fl3]: Not sure what is meant by this.

Commented [RC4R3]: Edited for clarity - what I meant was that the current study would inform the exact focus of the R01.

2. Research Strategy

1. Significance (no more than 1/2 page)

The importance of the problem is that four of five maternal deaths in the United States (U.S.) are preventable, and communication is a leading root cause of preventable maternal mortality.[1,10]~~[1,10]~~ Further, preventable maternal morbidity and mortality is a burden borne predominantly by Black women identify communication as a significant aspect of poor maternity care and outcomes.[1,26,27]~~[1,26,27]~~ A critical barrier to progress is that healthcare team members are frequently not aware of communication behaviors they engage in that are a barrier to developing the shared understanding crucial to high quality care and outcomes. The proposed project seeks to address this critical barrier through video-reflexive ethnography. Prior research has highlighted the importance of communication for maternity care quality and outcomes and has identified racially disparate communication as a critical area for intervention.[3]~~[3]~~ Indeed, many interventions have been developed to improve communication in inpatient maternity care.[18,19]~~[18,19]~~ A significant weakness in the rigor of the prior research, however, is a lack of interventions that 1) address racially disparate communication and 2) support clinicians in identifying – and changing – communication patterns that do not support the building of shared understanding.[18–20]~~[18–20]~~ The proposed project will improve scientific knowledge by develop

preliminary evidence as to whether VRE is a feasible and acceptable intervention to improve communication and address bias in communication in an inpatient maternity setting, as well as our understanding of how this method might support clinicians in developing a reflexive awareness of their communication patterns and biases. The proposed project will also improve clinical practice by inviting women and nurses to identify ways to improve communication. With these findings, we will be able to propose a trial with maternal morbidity as the primary outcome, and with a wider array of clinicians. If we find preliminary evidence that VRE holds the potential to significantly improve the development of shared understanding in healthcare by empowering clinicians and patients to identify and change their patterns of communication, this will be a remarkable step in moving the needle on maternity care quality and morbidity and mortality outcomes.

2. Innovation (no more than 1/2 page)

The proposed study challenges and seeks to shift the current research paradigm by focusing on nurses, communication, and a means of helping nurses become aware of and change their communication patterns to increase shared understanding as a means of addressing maternal morbidity and mortality in the U.S. This same work also seeks to shift current clinical practice paradigms in which nurses "talk at" patients at postpartum discharge and do not listen to them to understand the patient perspective. VRE disrupts the current paradigm by bringing into conscious awareness nurses' behaviors for reflection and deliberation. But importantly the video reveals patient responses to nurse behaviors and allows nurses to connect what they say and do to how patients respond. The application of VRE to address disparate communication in a healthcare setting is an innovative approach for helping clinicians develop self-awareness around their own communication patterns and a capacity for change. While VRE has been used to help White clinicians and educators develop an awareness of racially disparate communication,[23][23] the scaling-out of this intervention in a U.S. inpatient maternity setting to address nurse-patient communication, and racial disparities therein, is an innovative application.

3. Theory/Conceptual Framework (no more than 1/2 page)

Woodward et al.'s Health Equity Implementation Framework (Figure 1) is the conceptual framework that guides this work. For the proposed work, the clinical encounter of postpartum discharge teaching will be captured by video and the innovation is the process of VRE. Data will be collected on the clinical encounter, patient and provider factors, as well as the process of facilitating VRE (i.e., field notes, memos). There are patient and provider factors that will impact both the clinical encounter and how they interact with the

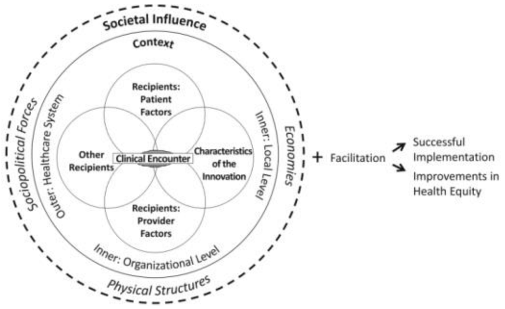

Figure 1: Health Equity Implementation Framework (Woodward, 2021)

innovation. We will assess this by inviting participants' feedback on the innovation and deploying a survey to gather information on demographics and nurse participants' perspectives on the feasibility, acceptability, and appropriateness of the innovation. We will also gather basic information on patients' medical and obstetric risk factors requiring targeted education during postpartum discharge teaching will be collected from the medical record. Other recipients might include family members who are present for discharge, but we are not including them in this study. This entire experience is impacted by the inner context of the Mother-Baby Unit and the outer context of the wider health system. The focus of the current work is on the clinical encounter and the patients/providers involved as a first step to determine feasibility and acceptability of this approach to improving patient/provider communication. Future work will expand this focus according to the conceptual framework to include organizational-level considerations and stakeholders.[28][28] We designed this study to be geared for implementation, so the qualitative and quantitative findings of the study will inform what might be needed to facilitate implementing VRE as an intervention, in addition to understanding how the VRE process might improve communication and equity therein.[28][28]

Formatted: Font: (Default) Arial, Font color: Black, , Superscript

Formatted: Font: (Default) Arial, Font color: Black, , Superscript

4. Design and Methods

The overall strategy and methodology of this study is to use a health equity-informed multi-methods qualitative study design to develop a deeper understanding of communication practices between nurses and patients during postpartum discharge teaching for first-time moms, including barriers and facilitators to communication, and recommendations for improvement. The planned analyses include qualitative analysis of video recordings of nurse-patient communication and audio/video recordings of patients and nurses reflecting on their communication interaction separately and then in separate focus groups (nurses only). While any participant may choose to stop being in the study at any time, we believe that the opportunity to review the interaction – even if it was non-supportive – should be offered to everyone so that they can choose whether or not they want the opportunity to reflect on the interaction. Provisions will be made in case revisiting the interaction is distressing (e.g., therapists, helpline, community support). Surveys will be deployed including basic sociodemographic data, and measures of intervention feasibility, appropriateness, and acceptability.

To accomplish Aims 1 and 2 we will conduct qualitative content analyses of video recordings of the VRE process, from the initial postpartum discharge teaching recording through the focus groups as detailed in Table 1. For Aim 3, we will use descriptive statistics to report survey results and qualitative content analysis to understand organizational leaders and nurses' thoughts about the feasibility, acceptability, appropriateness of VRE to improve nurse-patient communication during discharge postpartum teaching.

Formatted: Font: (Default) Arial

Formatted: Font: (Default) Arial

Formatted: Pattern: Clear (Background 1)

Formatted: Font: (Default) Arial

Table 1: Data Collection and Analysis Table[21][21]	
VRE Phases	Analysis Process
Pre-Data Collection	

Commented [MM5]: Should you say that this has already been done? That the equipment has been tested in the environment where the study will take place?

- Test whether OWLs (a smart webcam designed to create an immersive hybrid meeting experience in any space with the intelligent 360° conference camera, mic, and speaker that gets smarter over time) will sufficiently capture video and audio in postpartum rooms at hospital<br><br>- Conduct a "dry run" before proposed recording dates to understand logistical issues and plan accordingly	- This work has been completed.
Phase 1: Video Recording	~~First-level~~ Analysis
1.1 Nurses and patients will be consented prior to recording; we plan to over-sample women from racial/ethnic minority groups<br><br>1.2 Video record postpartum discharge communication between nurses and patients<br><br>1.3 After the recording, participants will be offered an opportunity to review the video right after recording for member checking[29] and will fill out a short sociodemographic survey (patient and clinician factors). ~~For Phase 2, they will be sent a link to separate sub folder with their video for confidentiality.~~<br><br>1.4 Edit each set of discharge instructions into separate clips<br><br>1.5 The presence of patient medical/obstetric factors influencing postpartum discharge teaching (e.g., hypertensive disorders, diabetes) will be abstracted from the chart	- Research team members will individually review each video as soon as possible after recording to ~~-~~ identify moments in the interaction that contain the richest or most interesting exchanges to take forward to Phase 2. "Richness" or "interesting" include communication breakdown, miscommunication, discordance body language that suggests disagreement or lack of shared understanding, as well as moments of effective communication and shared understanding. Further detail is provided in Data Collection below. ~~assure that communication between nurse and patient during discharge teaching is being captured~~.<br><br>- During ~~Team~~ meetings team members will discuss their individual opinions and come to consensus on which clips to take forward to Phase 2. ~~then choose vote on which moments in the interaction contain the richest or most interesting exchanges to take forward to Phase 2. "Richness" or "interesting" include communication breakdown, miscommunication, discordance body language that suggests disagreement or lack of shared understanding, as well as moments of effective communication and shared understanding.~~

Commented [MM6]: I know you borrowed a lot of what's in this table from my paper. It's been bugging me for a long time but I couldn't figure out why until now. You borrowed from a paper, not the grant. The grant didn't have anything like this in it. I think some of the confusion comes from what is expected in a grant vs. how a paper is structured. They are not the same. Consider taking out "first level" etc. because those words may be thought to correlate to first aim, etc. which they do not.

Formatted: Font: (Default) Arial, Font color: Black, Superscript

Commented [MM7]: Move this to the Phase 2 portion of the table

	- We will analyze dyads by racial concordance/discordance to see whether there are differences in communication<br><br>- Discharge communication will be reviewed for the presence of ~~specific~~ education~~teaching~~ specific to patient risk factors (e.g., information about follow-up testing for diabetes if patient had gestational diabetes) ~~related to personalized patient factors~~
Phase 2: Independent Review	~~Second-level~~ Analysis
2.1 Participants will be sent a link to separate sub folder with their video for confidentiality and ~~Participants~~ will independently review ~~a copy of the video-recorded conversation~~ selected clips of their postpartum discharge teaching interaction during a recorded video call<br><br>2.2 Participants will be encouraged to stop the video at any point and comment on their thoughts and feelings, what they were thinking at the time<br><br>2.3 Specific questions will be asked to prompt recall (e.g., Would you tell me more about what you were thinking when you looked away and focused on your phone?)<br><br>2.4 Comments will be edited into the video at the exact time stamp when the comments were made<br><br>2.5 Specific recommendations to improve the interaction specifically, and communication in general, will be solicited	- Team will watch the Phase 2 videos with embedded comments and discuss what we are viewing.<br><br>- The research team will use qualitative content analysis to develop findings.<br><br>The team will also look for similarities as well as differences in themes between the nurse and patient participants.<br><br>- Preliminary themes describing nurse-patient communication practices during postpartum discharge teaching (Aim 1)~~, including barriers and facilitators to effective communication (Aim 2),~~ will be identified via individual nurse and patient video reviews and discussed by the team.
Phase 3: ~~Focus Group~~Joint Review	~~Third-level~~ Analysis
3.1 Focus groups will be conducted by the study team members. There will be a focus group for patients and another for nurses. In these focus groups, patients and nurses will watch an aggregate of different discharge teaching video recordings with the	Transcripts of both focus groups will be analyzed using qualitative content analysis, looking for barriers and facilitators to effective communication (Aim 2), as well as similarities and differences across the two groups. ~~- Using qualitative content analysis a constant~~

Commented [MM8]: I don't know what this means. Are you saying that discharge teaching will be reviewed for evidence that the nurse included culturally relevant information specific to each patient? Considered each patient's unique learning style or preference for how information would be delivered?

Commented [fl9]: Themes of what? It is not clear what you are looking at? I am a little confused, Are aims 1, 2, 3 supposed to align with Phases 1, 2, and 3?

Commented [RC10R9]: Edited for clarity. At one point early on, I tried to align the Aims with the Phases and then realized it would be better separate.

embedded comments of both patients and nurses.. of patients and nurses independently with both sets of comments embedded<br><br>3.2 Participants will be asked to describe why they paused the video where they did to better understand the interaction from their point of view<br><br>3.2 We will ask participants to describe similarities and differences in communication during discharge teaching across the various video clips.<br><br>3.3 Next we will ask participants to identify barriers and facilitators to effective communication, that may have contributed to these similarities and differences.<br><br>3.43 To learn about the development of shared understanding, we will ask if comments from the independent review reveal information that was misunderstood or unknown at the time of the original communication	comparative technique, the research team will independently review the transcripts of both Phase 2 and Phase 3 reviews,<br><br>looking for similarities as well as differences in themes between the phases.<br><br>- Team will discuss findings to reach consensus.

Commented [MM11]: This doesn't fit here. Please delete

Sample Plan

The sample will consist of postpartum nurses who work in a large urban hospital, and the new mothers who receive education from them prior to discharge. To recruit nurses, we will 1) present the study at unit council meetings to get the word out and establish buy-in and 2) use nurses who are informal leaders and unit champions as recruiters. At the unit council meetings, we will share information about the work, gather input, address concerns, and answer questions. Information we are looking for includes: the best times of day for video recording, optimal placement of recording equipment in different room configurations, and how will a nurse know when a mom is about to be discharged (how much lead time will the nurse have). I will conduct study recruitment with the nurses, approaching nurses during unit council meetings and at other times identified as optimal by nursing leadership and the research nurses. We will hire two postpartum nurses, ideally one who will collect data on the night shift and one for the day shift, to be a part of the research team and work as research assistants. These two nurses will be either formal or informal leaders on the unit (e.g., opinion leaders, preceptors, or nurse educators), I will train them in their role as research assistants to consent participants and place

and retrieve video recording equipment. They will also participate in the data analysis, interpretation, and dissemination. We will recruit women during their postpartum stay, sharing information and inviting participation as early in their postpartum stay as possible. We will do this by developing a study flyer that is included with the admissions paperwork and posting the flyers in the shared lounge space on the postpartum units, as well as approaching women individually. There are 86 nurses on the unit and over 4,000 women give birth in the hospital annually. We are looking to record 20 dyads (one nurse and one mother) for VRE Phase 1. Prior research reported recruitment rates of 75% of nurses and 84% of physicians.[21] as we expect attrition for VRE Phase 2 based on prior work and want to ensure at least 10 dyads for VRE Phase 2. We will purposefully sample dyads who are racially concordant as well as those who are racially discordant. Initially we will try for 10 of each but may adapt the strategy as we go.

Overall, we are collecting audio-video and survey data. In Aim 1, a communication interaction (postpartum discharge teaching) is video recorded. Moments in the interaction that are particularly interesting (e.g., a moment of tension, missed communication, etc.) are clipped for viewing by the research team. In Aim 2, participants in the Phase 1 interaction view the clips independently. They are encouraged to pause at different moments in the conversation to share what they were thinking or feeling as the interaction happened, or what they are currently thinking or feeling. These reflections are inserted into the original clips at the exact time stamp they were made. In Aim 3, we will run focus groups of the nurse participants in which they will watch edited videos and be encouraged to share why they paused the video where they did to understand their reactions, as well as to reflect on how their understanding has changed based on what the other participant shared. These focus groups will also be video recorded. We will also have short sociodemographic survey data and a separate short survey for nurses regarding the feasibility, acceptability, and appropriateness of VRE as an intervention.[2828]

Data Collection Activities

Please see Table 1 for details on study procedures and analysis. Prior to collecting data, we will have two dry-run days on the unit in which the research team will observe the postpartum nurses' workflow over the course of the day to understand when best to consent them and the women. We will also examine the postpartum discharge rooms to determine the best placement for the OWLs. Postpartum discharge instructions might be conducted at any time, though typically they'll be conducted in late morning/early afternoon or the evening prior to discharge. We will check in early with nurses on recording days about when they foresee doing postpartum discharge instructions and ask them to text or alert the study nurse when ready to do the teaching.

During the data collection phase, after informed consent has been obtained, the research team nurse will place an OWL in the room in a predetermined optimal position and then leave the room. After postpartum discharge teaching is complete, the research team nurse will collect the OWL, collect a short socio-demographic survey, and provide the nurse and the patient with

Formatted: Font: (Default) Arial, Font color: Black, Superscript

Commented [fl12]: How many nurses work on this unit, and do you have enough to sample from to achieve your recruitment goal?

Commented [RC13R12]: There are 87 nurses on the unit, so we are looking to recruit about 23% initially so that we can have about 11.5% of the population in Phase 2.

Commented [MM14R12]: Just provide numbers: 87 work on the unit and we aim to recruit 20 of them. You might want to cite other literature that shows similar recruitment rates for other VRE studies.

Commented [fl15]: In the table above, it says you will run focus groups with both nurses and patients.

Commented [RC16R15]: Thank you - edited.

Commented [fl17]: You have so much redundant information between the table, this paragraph, and the following section. In the future, I would recommend being more succinct. When you repeat information you run the risk of inconsistencies, as below.

Commented [RC18R17]: Thank you - this is a good recommendation!

Commented [MM19R17]: But you haven't taken the recommendation! Consider deleting this paragraph and add one sentence that refers the reader to Table 1 that provides details on study procedures.

Formatted: Font: (Default) Arial, Font color: Black, Superscript

Commented [MM20]: This belongs in methods, not potential challenges

follow-up information for VRE Phase 2. Data from the OWLs will be uploaded onto a secure research drive. The research team will watch the video and edit for the richest moments in the interaction. For example, we will look for moments where teaching is personalized or seems generic, and for moments of shared-decision making, autonomy supportive consultation, or assertion-acquiescence.[30] We will also look for the presence of medical jargon,[31,32] or lack thereof, in explanations, and for the sharing of warning signs and symptoms, and the presentation of infant care information.[33,34] We will look for the presence or absence of teach back after the sharing of information,[35] as well as moments of question asking and how those questions are responded to.[36,37] We will also look for nonverbal cues, including making eye contact, repeating back information (without being asked to), agreeing or disagreeing, body language that would suggest openness to what is being said, or alternatively discomfort, etc.[38] We will then, for VRE Phase 2, schedule virtual meetings with each patient and nurse in which a member of the research team will watch the video with the participant. These meetings will be recorded so that a video and a transcript are generated. This information will also be saved to the secure research drive. ~~These videos will again be edited for the richest moments in reflection and recommendations regarding communication.~~ During VRE Phase 2 (independent review) with the patient participants, we will also ask for their recommendations for interventions to improve nurse-patient communication practices. For VRE Phase 3 (joint review), one of the team members, who is a qualitative methods expert, will facilitate separate focus groups for nurses and patients~~of the nurse participants~~ who will watch selected~~the edited~~ Phase 2 videos of interactions with ~~and f~~feedback. Phase 3 participants will be encouraged to ~~in order to~~ generate ~~their own~~ recommendations to improve nurse-patient communication practices. We will ask nurses to rate the feasibility, acceptability, and appropriateness of VRE as an intervention to improve nurse-patient communication via a questionnaire of three validated short scales.[39]~~[29]~~ These joint review sessions will be recorded with an OWL if conducted in-person and via the teleconferencing platform if conducted virtually. ~~They will also be asked to rate the feasibility, acceptability, and efficacy of VRE as an intervention to improve nurse-patient communication.~~

We will use a qualitative content analysis approach to analyzing the video data. The research team will keep an audit document to track their analytic decisions and will debrief to discuss interpretive biases. ~~Feasibility, acceptability, and appropriateness will be assessed via validated surveys.~~[39]~~[29]~~ ~~We will also ask for nurses' recommendations about how to address perceptions of risk for future participants (e.g., what circumstances might make participation feel like less of a risk and their suggestions to address risk).~~ Descriptive statistics will be used to report the survey data and sociodemographic information.

~~The clinical director for women's health at the hospital is enthusiastic about this project and the nurse manager, assistant nurse manager, and nurse educator for the postpartum unit are also supportive of the project and excited to see it accomplished. One of the co-investigators on the project is a Maternal-Fetal Medicine physician at the hospital. I will work with her to confirm organization- and provider-level support to conduct this study, including presenting the study at the Maternal-Fetal Medicine Research Meeting. I attended all of the Mother-Baby Unit staff~~

Formatted: Font: (Default) Arial, Font color: Black, Superscript

Formatted: Font: (Default) Arial, Font color: Black, Superscript

Formatted: Font: (Default) Arial, Font color: Black, Superscript

Formatted: Font: (Default) Arial, Font color: Black, Superscript

Formatted: Font: (Default) Arial, Font color: Black, Superscript

Formatted: Font: (Default) Arial, Ligatures: None

Formatted: Font: (Default) Arial, Ligatures: None

Formatted: Font: (Default) Arial, Ligatures: None

Formatted: Font: (Default) Arial, Font color: Black, Superscript

~~meetings in September 2023 to introduce the study and recruit two nurses to the research team. I will attend the staff meetings again after receiving IRB approval to review the study and invite participation.~~

Commented [fl21]: This paragraph is out of place, should not be in methods.

Potential Challenges, Alternative Strategies, and Benchmarks for Success

Video recording clinical interactions is a sensitive subject. To address this, we have met with clinical nursing leadership at the hospital ~~and have the enthusiastic support of the clinical director, nurse manager, assistant nurse manager, and nurse educator. The leadership not only support the research, and are enthusiastic about its application, but~~ who strongly agree with the research team that the videos will not be used to penalize nurses. We will ~~also~~ ask for nurses' recommendations about how to address perceptions of risk of being penalized for future participants (e.g., what circumstances might make participation feel like less of a risk and their suggestions to address risk). As a result, findings will only be shared with the leadership team in deidentified aggregate. We also plan to engage with staff nurses early and often, coming to unit councils at the invitation of leadership to share about the study and offer chances to engage with the researchers and ask questions. We also plan to hire ~~two~~ one postpartum nurse~~s~~ to be a study team member who will become a familiar presence during the study.~~s and will look to them – and potentially other interested nurses –~~ We will encourage other interested nurses to champion the work to their peers.

~~Postpartum discharge instructions might be conducted at any time, though typically they'll be conducted in late morning/early afternoon or the evening prior to discharge. We will check in early with nurses on recording days about when they foresee doing postpartum discharge instructions and ask them to text or alert the study nurse when ready to do the teaching.~~

Commented [MM22]: This belongs in methods, not potential challenges

The Hawthorne effect is frequently mentioned as a concern, though prior research indicates that many participants eventually stop paying attention to the video in the room.[40–42]~~[30–32]~~ To address this challenge, we will try to use an OWL, which has a smaller and less obtrusive footprint. We will also ask participants about their awareness of being videorecorded ~~taped~~ as part of the post-video survey. Also, prior work found that it was important to minimize the time gap between ~~Phases 1 and~~ initial video recording and independent review.[21] To address this, we will schedule indepdent reviews (Phase 2 meetings) with patients and nurses when their interaction is recorded with the goal of having that Phase 2 meeting within a week.~~aim to set up Phase 2 meetings at the conclusion of Phase 1.~~

Formatted: Font: (Default) Arial, Font color: Black, Superscript

Commented [MM23]: No. what this says is that independent review won't start until all videos have been recorded. I think you want to shorten the time between video recording and independent review

To assess the feasibility of this work, we engaged clinical leadership both as team members and as advocates for the project. With regards to ongoing feasibility, we will ask participants to complete a feasibility survey as part of the post-recording data collection. ~~One of the most high-risk aspects of this work is the risk of being penalized – whether as a nurse by one's supervisors and peers, or as a patient by one's healthcare team.~~ We plan to incentivize recruitment by ~~reimbursing~~ offering small tokens of appreciation for participation ($xx gift cards to both nurses and patients), will offer to protect confidentiality by blurring faces.~~,~~ ~~and will protect against~~

Commented [MM24]: You've already talked about risk,, so move this to the discussion of risk or delete. I think it is duplicative

~~penalization by only reporting results in deidentified aggregate.~~ Benchmarks for success include: recruiting ~~10~~ 20 dyads; completion of phases per timeline; having at least ~~7~~ 10 dyads complete all three phases; and dissemination per timeline. Further, success for this project includes the sharing of actionable recommendations for change with the unit that the nurse participants "own" and the development of the MBU research nurses' capacity in research.

We have considered the implications of hiring two postpartum nurses to be on the study team. This idea was endorsed by the clinical director and unit leadership. We plan to hire one staff nurse to be on the study team due to their content expertise and knowledge of the unit and its culture. We will also hire a research coordinator from the Women's Health Research Center staff at Penn Medicine due to their research and recruitment expertise. The nurse on the research team will not be a study participant.

5. Preliminary Studies

Maternity nursing, outcomes, and equity research

The PI's current program of research focuses on maternity nursing – and the organizational resources which support nursing care – as levers to improve maternity care quality, outcomes, and equity therein. This work includes understanding how variation in maternity nursing resources – like the work environment and staffing – are associated with high-performing hospitals where in Black women have appropriately low low-risk cesarean rates. As a result of this work, as well as her clinical experience and nurse scientist role, the PI has a deep understanding of hospital organizational resources, unit functioning, and stakeholders, that will be important for implementing the current work.

Communication and Equitable care and outcomes

The PI has two ongoing qualitative studies focused on communication, ~~and~~ equitable care, and outcomes. In one, the PI is using ~~In the first of these,~~ incident reports from inpatient maternity units ~~in a hospital are being used~~ to understand types of communication failure ~~types~~, how these vary~~ied~~ across patient race/ethnicity, and how they are~~were~~ associated with patient harm and severe maternal morbidity. In the other study, the PI is studying how women living with HIV who have recently had children perceive provider communication about their infant feeding choices~~The second study women living with HIV's perceptions of provider communication around infant feeding choices~~. Accordingly, the PI has experience studying healthcare communication and equity in the perinatal space.

Qualitative research

In addition to the two studies mentioned in the preceding paragraph, another study explores maternity nurses' perceptions of what helps or hinders them in providing quality care to vulnerable populations. This study identifies systemic and personal barriers and facilitators, including urgent points of intervention, related to providing high quality, equitable care for women giving birth in hospitals.

Commented [MM25]: Unnecessary. Already mentioned

Commented [MM26]: No. don't put this on the nurses who may not feel safe in speaking up or commenting on something unrelated to their work. You have to show that you are responsive to the feedback. I would do what we discussed during our last call and that is that one staff nurse be on the study team b/c of content expertise and knowledge of the unit and its culture. The other should come form the WHRC b/c of research expertise.

Commented [fl27]: I don't understand this sentence.

Commented [RC28R27]: Edited for clarity.

6. Readiness to Launch Project

Readiness Table	
Activity	Status
Site(s) secured	Study will occur in the Mother-Baby Unit at Pennsylvania Hospital. Approval to conduct the study has been given by nursing and physician stakeholders. ~~Nursing leadership supportive; Physician leadership supportive and lent support of administrative director for IRB submission; pe~~nding IRB approval. ~~IRB approval received.~~
Stakeholder engagement	Have met with nursing leadership; attended nursing staff meetings in September 2023 and physician stakeholder group meeting in October 2023. Have received nursing and physician stakeholder approval at the department, hospital and system-level (Women's Health Clinical Director of Nursing, System-level Obstetric Physician approval, MBU Nurse Manager approval, CNO approval. ~~IRB approval~~).
Collaborators engaged	Research assistant hired; hiring of one Mother-Baby Unit nurses to the research team in process; patient collaborator has received approval from her employer and her paperwork has been entered into the Penn system. Plan for initial team meeting in January 2023.~~finalization of patient collaborator in process; plan for initial team meeting December 2023~~
Expertise needed to accomplish project activities, study personnel with this expertise	Conducting focus groups (Dr. Patrina Sexton Topper, co-I), VRE (Dr. Milisa Manojlovich, research mentor), qualitative content analysis (multiple team members)
Recruitment strategies in place with appropriate site agreements	Recruitment strategies in place, reviewed and recommendations made by nursing and physician group; pending IRB approval
IRB application	~~IRB application developed, submitted, and now in revision for resubmission before December 2023.~~IRB approval (Protocol #854936) received on 12/6/23~~[date]~~ .

Commented [MM29]: This doesn't get at "site secured." Consider saying something like, "Study will occur in the xx bed Mother Baby Unit at XX hospital." Approval to conduct study here has been given by all stakeholders (nursing and physician leadership).

Commented [MM30]: Spell out

Commented [MM31]: Include IRB approval #.

7. References (not included in eight-page limit)

1. Hoyert D. *Maternal Mortality Rates in the United States, 2020*.; 2022. doi:https://dx.doi.org/10.15620/cdc:113967

2. Trost S, Beauregard J, Chandra G, et al. *Pregnancy-Related Deaths: Data from Maternal Mortality Review Committees in 36 US States, 2017-2019*.; 2017.

3. National Academies of Sciences, Engineering and M. *Advancing Maternal Health Equity and Reducing Maternal Morbidity and Mortality: Proceedings of a Workshop*. The National Academies Press; 2021. doi:10.17226/26307

4. Attanasio L, Kozhimannil KB. Patient-reported Communication Quality and Perceived Discrimination in Maternity Care. *Med Care*. 2015;53(10):863-871. doi:10.1097/MLR.0000000000000411

5. McLemore MR, Altman MR, Cooper N, Williams S, Rand L, Franck L. Health care experiences of pregnant, birthing and postnatal women of color at risk for preterm birth. *Soc Sci Med*. 2018;201(January):127-135. doi:10.1016/j.socscimed.2018.02.013

6. Janevic T, Piverger N, Afzal O, Howell EA. "Just because you have ears doesn't mean you can hear" - perception of racial-ethnic discrimination during childbirth. *Ethn Dis*. 2020;30(4):533-542. doi:10.18865/ED.30.4.533

7. Brantley MD, Callaghan W, Cornell A, et al. *Report from Nine Maternal Mortality Review Committees*.; 2018. http://reviewtoaction.org/Report_from_Nine_MMRCs

8. Brennan RA, Keohane CA. How Communication Among Members of the Health Care Team Affects Maternal Morbidity and Mortality. *JOGNN - Journal of Obstetric, Gynecologic, and Neonatal Nursing*. 2016;45(6):878-884. doi:10.1016/j.jogn.2016.03.142

9. Sentinel Event Alert 30: Preventing infant death and injury during delivery | The Joint Commission. Accessed March 17, 2020. https://www.jointcommission.org/resources/patient-safety-topics/sentinel-event/sentinel-event-alert-newsletters/sentinel-event-alert-issue-30-preventing-infant-death-and-injury-during-delivery/

10. Pettker CM, Grobman WA. Clinical Expert Series Obstetric Safety and Quality. *Obstet Gynecol*. 2015;126:196-206. doi:10.1097/AOG.0000000000000918

11. Bajaj K de RAGDAPNo 20(21) 0040 6 EF. *The Contribution of Diagnostic Errors to Maternal Morbidity and Mortality During and Immediately After Childbirth: State of the Science*.; 2021. www.ahrq.gov

12. Altman MR, Oseguera T, McLemore MR, Kantrowitz-Gordon I, Franck LS, Lyndon A. Information and power: Women of color's experiences interacting with health care providers in pregnancy and birth. *Soc Sci Med*. 2019;238(October 2018):112491. doi:10.1016/j.socscimed.2019.112491

13. Wang E, Glazer KB, Sofaer S, Balbierz A, Howell EA. Racial and Ethnic Disparities in Severe Maternal Morbidity: A Qualitative Study of Women's Experiences of Peripartum Care. *Women's Health Issues*. 2021;31(1):75-81. doi:10.1016/j.whi.2020.09.002

14. Slaughter-Acey JC, Caldwell CH, Misra DP. The Influence of Personal and Group Racism on Entry Into Prenatal Care Among-African American Women. Published online 2013. doi:10.1016/j.whi.2013.08.001

15. Chambers BD, Arega HA, Arabia SE, et al. Black Women's Perspectives on Structural Racism across the Reproductive Lifespan: A Conceptual Framework for Measurement Development. *Matern Child Health J*. 2021;25(3):402-413. doi:10.1007/s10995-020-03074-3

16. Chambers BD, Taylor B, Nelson T, et al. Clinicians' Perspectives on Racism and Black Women's Maternal Health. *Women's Health Reports*. 2022;3(1):476-482. doi:10.1089/whr.2021.0148

17. Sun M, Oliwa T, Peek ME, Tung EL. Negative Patient Descriptors: Documenting Racial Bias In The Electronic Health Record. *Health Aff*. 2022;41(2):203-211. doi:10.1377/hlthaff.2021.01423

18. Lippke S, Wienert J, Keller FM, et al. Communication and patient safety in gynecology and obstetrics - Study protocol of an intervention study. *BMC Health Serv Res*. 2019;19(1):1-18. doi:10.1186/s12913-019-4579-y

19. Chang YS, Coxon K, Portela AG, Furuta M, Bick D. Interventions to support effective communication between maternity care staff and women in labour: A mixed-methods systematic review. *Midwifery*. 2018;59(December 2017):4-16. doi:10.1016/j.midw.2017.12.014

20. Aggarwal R, Plough A, Henrich N, et al. The design of "TeamBirth": A care process to improve communication and teamwork during labor. *Birth*. 2021;(October 2020):1-7. doi:10.1111/birt.12566

21. Manojlovich M, Frankel RM, Harrod M, et al. Formative evaluation of the video reflexive ethnography method, as applied to the physician-nurse dyad. *BMJ Qual Saf*. 2019;28(2):160-166. doi:10.1136/bmjqs-2017-007728

22. Korstjens I, Mesman J, van Helmond I, de Vries R, Nieuwenhuijze M. The paradoxes of communication and collaboration in maternity care: A video-reflexivity study with professionals and parents. *Women and Birth*. 2021;34(2):145-153. doi:10.1016/j.wombi.2020.01.014

23. Campbell KH, Valauri A. Our Voices Matter: Using Video-Cued Ethnography to Facilitate a Conversation about Race between Parents of Color and Preservice Teachers. *Anthropol Educ Q*. 2019;50(3):333-339. doi:10.1111/aeq.12296

24. Neuwirth EB, Bellows J, Jackson AH, Price PM. How Kaiser Permanente uses video ethnography of patients for quality improvement, such as in shaping better care transitions. *Health Aff*. 2012;31(6):1244-1250. doi:10.1377/hlthaff.2012.0134

25. McHugh S, Sheard L, O'Hara J, Lawton R. The feasibility and acceptability of implementing video reflexive ethnography (VRE) as an improvement tool in acute maternity services. *BMC Health Serv Res*. 2022;22(1). doi:10.1186/s12913-022-08713-9

26. Janevic T, Piverger N, Afzal O, Howell EA. "Just Because You Have Ears Doesn't Mean You Can Hear"-Perception of Racial-Ethnic Discrimination During Childbirth. *Ethn Dis*. 2020;30(4):533-542. doi:10.18865/ed.30.4.533

27. Dahlem CHY, Villarruel AM, Ronis DL. African American Women and Prenatal Care: Perceptions of Patient–Provider Interaction. *West J Nurs Res*. 2015;37(2):217-235. doi:10.1177/0193945914533747

28. Weiner BJ, Lewis CC, Stanick C, et al. Psychometric assessment of three newly developed implementation outcome measures. *Implementation Science*. 2017;12(1). doi:10.1186/s13012-017-0635-3

29. Birt L, Scott S, Cavers D, Campbell C, Walter F. Member Checking: A Tool to Enhance Trustworthiness or Merely a Nod to Validation? *Qual Health Res*. 2016;26(13):1802-1811. doi:10.1177/1049732316654870

30. Jackson C, Land V, Holmes EJB. Healthcare professionals' assertions and women's responses during labour: A conversation analytic study of data from One born every minute. *Patient Educ Couns*. 2017;100(3):465-472. doi:10.1016/j.pec.2016.10.004

31. Lerner EB, Jehle DVK, Janicke DM, Moscati RM. Medical communication: Do our patients understand? *American Journal of Emergency Medicine*. 2000;18(7):764-766. doi:10.1053/ajem.2000.18040

32. Blee S, Rosenberg B, Switchenko JM, et al. Understanding immunotherapy terminology: An analysis of provider-patient conversations. *ImmunoMedicine*. 2021;1(2). doi:10.1002/imed.1028

33. Sword W, Watt S. Learning needs of postpartum women: Does socioeconomic status matter? *Birth*. 2005;32(2):86-92. doi:10.1111/j.0730-7659.2005.00350.x

34. Persson EK, Fridlund B, Kvist LJ, Dykes AK. Mothers' sense of security in the first postnatal week: Interview study. *J Adv Nurs*. 2011;67(1):105-116. doi:10.1111/j.1365-2648.2010.05485.x

35. Graham S, Brookey J. Do Patients Understand? *Perm J*. 2008;12(3):67-69. www.npsf.org/pchc/index.php

36. Hilder J, Stubbe M, MacDonald L, Abels P, Dowell AC. Communication in high risk ante-natal consultations: A direct observational study of interactions between patients and obstetricians. *BMC Pregnancy Childbirth*. 2020;20(1). doi:10.1186/s12884-020-03015-6

37. Martin L, Gitsels-van der Wal JT, Pereboom MTR, Spelten ER, Hutton EK, van Dulmen S. Midwives' perceptions of communication during videotaped counseling for prenatal anomaly tests: How do they relate to clients' perceptions and independent observations? *Patient Educ Couns*. 2015;98(5):588-597. doi:10.1016/j.pec.2015.02.002

38. Hamel LM, Moulder R, Ramseyer FT, et al. Nonverbal Synchrony: An Indicator of Clinical Communication Quality in Racially-Concordant and Racially-Discordant Oncology Interactions. *Cancer Control*. 2022;29. doi:10.1177/10732748221113905

39. Weiner BJ, Lewis CC, Stanick C, et al. Psychometric assessment of three newly developed implementation outcome measures. *Implementation Science*. 2017;12(1). doi:10.1186/s13012-017-0635-3

40. Themessl-Huber M, Humphris G, Dowell J, Macgillivray S, Rushmer R, Williams B. Audio-visual recording of patient-GP consultations for research purposes: A literature review on recruiting rates and strategies. *Patient Educ Couns*. 2008;71(2):157-168. doi:10.1016/j.pec.2008.01.015

41. Henry SG, Fetters MD. Video elicitation interviews: A qualitative research method for investigating physician-patient interactions. *Ann Fam Med*. 2012;10(2):118-125. doi:10.1370/afm.1339

42. Paradis E, Sutkin G. Beyond a good story: From Hawthorne Effect to reactivity in health professions education research. *Med Educ*. 2017;51(1):31-39. doi:10.1111/medu.13122

1. Hoyert D. *Maternal Mortality Rates in the United States, 2020*. 2022. doi:https://dx.doi.org/10.15620/cdc:113967. Accessed at https://www.cdc.gov/nchs/data/hestat/maternal-mortality/2020/maternal-mortality-rates-2020.htm

2. Trost S, Beauregard J, Chandra G, et al. *Pregnancy-Related Deaths: Data from Maternal Mortality Review Committees in 36 US States, 2017-2019*. 2017. Accessed at https://www.cdc.gov/reproductivehealth/maternal-mortality/erase-mm/data-mmrc.html

3. National Academies of Sciences, Engineering and Medicine. *Advancing Maternal Health Equity and Reducing Maternal Morbidity and Mortality: Proceedings of a Workshop*. The National Academies Press; 2021. doi:10.17226/26307

4. Attanasio L, Kozhimannil KB. Patient-reported Communication Quality and Perceived Discrimination in Maternity Care. *Med Care*. 2015;53(10):863-871. doi:10.1097/MLR.0000000000000411

5. McLemore MR, Altman MR, Cooper N, Williams S, Rand L, Franck L. Health care experiences of pregnant, birthing and postnatal women of color at risk for preterm birth. *Soc Sci Med*. 2018;201(January):127-135. doi:10.1016/j.socscimed.2018.02.013

6. Janevic T, Piverger N, Afzal O, Howell EA. “Just because you have ears doesn’t mean you can hear" - perception of racial-ethnic discrimination during childbirth. *Ethn Dis*. 2020;30(4):533-542. doi:10.18865/ED.30.4.533

7. Brantley MD, Callaghan W, Cornell A, et al. *Report from Nine Maternal Mortality Review Committees*. 2018. Accessed at http://reviewtoaction.org/Report_from_Nine_MMRCs

8. Brennan RA, Keohane CA. How Communication Among Members of the Health Care Team Affects Maternal Morbidity and Mortality. *JOGNN*. 2016;45(6):878-884. doi:10.1016/j.jogn.2016.03.142

9. Joint Commission. Sentinel Event Alert 30: Preventing infant death and injury during delivery. Accessed at https://www.jointcommission.org/resources/patient-safety-topics/sentinel-event/sentinel-event-alert-newsletters/sentinel-event-alert-issue-30-preventing-infant-death-and-injury-during-delivery/

10. Pettker CM, Grobman WA. Clinical Expert Series Obstetric Safety and Quality. *Obstet Gynecol*. 2015;126:196-206. doi:10.1097/AOG.0000000000000918

11. Bajaj K, de Roche A, Goffman D. *The Contribution of Diagnostic Errors to Maternal Morbidity and Mortality During and Immediately After Childbirth: State of the Science*. Rockville, MD: Agency for Healthcare Research and Quality; September 2021. AHRQ Publication No. 20(21)-0040-6-EF.

12. Altman MR, Oseguera T, McLemore MR, Kantrowitz-Gordon I, Franck LS, Lyndon A. Information and power: Women of color's experiences interacting with health care providers in pregnancy and birth. *Soc Sci Med*. 2019;238:112491. doi:10.1016/j.socscimed.2019.112491

13. Wang E, Glazer KB, Sofaer S, Balbierz A, Howell EA. Racial and Ethnic Disparities in Severe Maternal Morbidity: A Qualitative Study of Women's Experiences of Peripartum Care. *Women's Health Issues*. 2021;31(1):75-81. doi:10.1016/j.whi.2020.09.002

14. Slaughter-Acey JC, Caldwell CH, Misra DP. The Influence of Personal and Group Racism on Entry Into Prenatal Care Among African American Women. *Womens Health Issues*. 2013;23(6):e381-e387. doi:10.1016/j.whi.2013.08.001

15. Chambers BD, Arega HA, Arabia SE, et al. Black Women's Perspectives on Structural Racism across the Reproductive Lifespan: A Conceptual Framework for Measurement Development. *Matern Child Health J*. 2021;25(3):402-413. doi:10.1007/s10995-020-03074-3

16. Chambers BD, Taylor B, Nelson T, et al. Clinicians' Perspectives on Racism and Black Women's Maternal Health. *Women's Health Reports*. 2022;3(1):476-482. doi:10.1089/whr.2021.0148

17. Sun M, Oliwa T, Peek ME, Tung EL. Negative Patient Descriptors: Documenting Racial Bias In The Electronic Health Record. *Health Aff*. 2022;41(2):203-211. doi:10.1377/hlthaff.2021.01423

18. Lippke S, Wienert J, Keller FM, et al. Communication and patient safety in gynecology and obstetrics - Study protocol of an intervention study. *BMC Health Serv Res*. 2019;19(1):1-18. doi:10.1186/s12913-019-4579-y

19. Chang YS, Coxon K, Portela AG, Furuta M, Bick D. Interventions to support effective communication between maternity care staff and women in labour: A mixed-methods systematic review. *Midwifery*. 2018;59:4-16. doi:10.1016/j.midw.2017.12.014

20. Aggarwal R, Plough A, Henrich N, et al. The design of "TeamBirth": A care process to improve communication and teamwork during labor. *Birth*. 2021;(October 2020):1-7. doi:10.1111/birt.12566

21. Manojlovich M, Frankel RM, Harrod M, et al. Formative evaluation of the video reflexive ethnography method, as applied to the physician-nurse dyad. *BMJ Qual Saf*. 2019;28(2):160-166. doi:10.1136/bmjqs-2017-007728

22. Korstjens I, Mesman J, van Helmond I, de Vries R, Nieuwenhuijze M. The paradoxes of communication and collaboration in maternity care: A video-reflexivity study with professionals and parents. *Women and Birth*. 2021;34(2):145-153. doi:10.1016/j.wombi.2020.01.014

23. Campbell KH, Valauri A. Our Voices Matter: Using Video-Cued Ethnography to Facilitate a Conversation about Race between Parents of Color and Preservice Teachers. *Anthropol Educ Q*. 2019;50(3):333-339. doi:10.1111/aeq.12296

24. Neuwirth EB, Bellows J, Jackson AH, Price PM. How Kaiser Permanente uses video ethnography of patients for quality improvement, such as in shaping better care transitions. *Health Aff*. 2012;31(6):1244-1250. doi:10.1377/hlthaff.2012.0134

25. McHugh S, Sheard L, O'Hara J, Lawton R. The feasibility and acceptability of implementing video reflexive ethnography (VRE) as an improvement tool in acute maternity services. *BMC Health Serv Res*. 2022;22(1). doi:10.1186/s12913-022-08713-9

26. Janevic T, Piverger N, Afzal O, Howell EA. "Just Because You Have Ears Doesn't Mean You Can Hear"-Perception of Racial-Ethnic Discrimination During Childbirth. *Ethn Dis*. 2020;30(4):533-542. doi:10.18865/ed.30.4.533

27. Dahlem CHY, Villarruel AM, Ronis DL. African American Women and Prenatal Care: Perceptions of Patient–Provider Interaction. *West J Nurs Res*. 2015;37(2):217-235. doi:10.1177/0193945914533747

28. Weiner BJ, Lewis CC, Stanick C, et al. Psychometric assessment of three newly developed implementation outcome measures. *Implementation Science*. 2017;12(1). doi:10.1186/s13012-017-0635-3

29. Weiner BJ, Lewis CC, Stanick C, et al. Psychometric assessment of three newly developed implementation outcome measures. *Implementation Science*. 2017;12(1). doi:10.1186/s13012-017-0635-3

30. Themessl-Huber M, Humphris G, Dowell J, Macgillivray S, Rushmer R, Williams B. Audio-visual recording of patient-GP consultations for research purposes: A literature review on recruiting rates and strategies. *Patient Educ Couns*. 2008;71(2):157-168. doi:10.1016/j.pec.2008.01.015

31. Henry SG, Fetters MD. Video elicitation interviews: A qualitative research method for investigating physician-patient interactions. *Ann Fam Med*. 2012;10(2):118-125. doi:10.1370/afm.1339

32. Paradis E, Sutkin G. Beyond a good story: From Hawthorne Effect to reactivity in health professions education research. *Med Educ*. 2017;51(1):31-39. doi:10.1111/medu.13122

Formatted: Indent: Hanging: 0.44", Space Before: 0 pt, After: 8 pt, Line spacing: Multiple 1.08 li, Don't adjust space between Latin and Asian text, Don't adjust space between Asian text and numbers, Pattern: Clear
